# Characterization of Vasorelaxant Principles from the Needles of *Pinus morrisonicola* Hayata

**DOI:** 10.3390/molecules23010086

**Published:** 2017-12-31

**Authors:** Guan-Heng Chen, Yue-Chiun Li, Nan-Hei Lin, Ping-Chung Kuo, Jason T. C. Tzen

**Affiliations:** 1 Graduate Institute of Biotechnology, National Chung-Hsing University, Taichung 402, Taiwan; bnbn23005856@hotmail.com (G.-H.C.); ycli0126@gmail.com (Y.-C.L.); CMNHEI@mohw.gov.tw (N.-H.L.); 2 School of Pharmacy, College of Medicine, National Cheng Kung University, Tainan 701, Taiwan

**Keywords:** *Pinus morrisonicola*, lignan, acylated flavonoid glycoside, voltage-operated calcium channel, antihypertensive

## Abstract

*Pinus morrisonicola* Hayata, usually called Taiwan five-leaf pine (5LP), is an endemic species in Taiwan and is traditionally used to relieve hypertension symptoms and improve cardiovascular function. In this study, the needle extract of 5LP was fractionated and analyzed by LC/MS/MS to search for possible antihypertensive candidates. In addition, bioassay-guided purification of the bioactive components was performed by Ca^2+^ fluorescent signal (Fluo 4-AM) assays. Two dihydrobenzofuran lignans, pinumorrisonide A (**1**) and icariside E4 (**2**), and one acylated flavonoid glycoside, kaempferol 3-*O*-α-(6‴-*p*-coumaroylglucosyl-β-1,4-rhamnoside) (**3**) were characterized from the active fractions. The structure of a new compound **1** was established on the basis of 2D NMR spectroscopic and mass spectrometric analyses, and the known compounds **2** and **3** were identified by comparison of their physical and spectroscopic data with those reported in the literature. The purified compounds **1**–**3** exhibited significant inhibition of Ca^2+^ fluorescence with IC_50_ values of 0.71, 0.36, and 0.20 mM, respectively. A mechanism study showed that these compounds showed vasorelaxant effects by blocking the voltage-operated Ca^2+^ channel (VOCC) and inhibiting Ca^2+^ influx to the cytoplasmic. These results suggested that 5LP and the three characterized components could be promising antihypertensive candidates for the use as VOCC blockers.

## 1. Introduction

*Pinus morrisonicola* Hayata is an endemic species widely distributed in the mountain areas of Taiwan, and trivially named as five-leaf pine (5LP). The needle of 5LP is locally used to generate folk medicine and functional food in Taiwan, e.g., directly extracted with water to prepare an instant tonic drink or typically fermented in a time-consuming process to generate liquor and vinegar products [1,2]. It is believed to have many pharmacological effects, such as anti-aging, anti-hypercholesterolemia and anti-hypertension. It was reported that the water extract of 5LP inhibits proliferation and promotes apoptosis of human promyelocytic leukemia cells and exhibited strong cytotoxic effects on GBM8901 glioblastoma cells [3,4]. Moreover, the ethyl acetate fraction and the ethanol extract of pine needles were found to contain large amounts of polyphenols and flavonoids, and have been shown to possess strong free radical scavenging ability [5].

Hypertension has gained much more attention than any other cardiovascular syndromes due to the acceleration of urbanization. Some scholars estimate that in 2025, the total number of adults with hypertension will be 1.56 billion [6]. Hypertension not only affects subsequent mortality rates, but also increases the risk of getting heart stroke, coronary disease and chronic kidney disorders [7,8]. Despite many different types of antihypertensive drugs been used in therapeutic applications, Ca^2+^ channel blockers (CCBs) have been in the first line of treatment for more than 25 years [8]. Three major types of CCB drugs are diltiazem (a benzothiazepinone, BTZ), nifedipine (a 1,4 dihydropyridine, DHP) and verapamil (a phenylalkylamine, PAA) [9]. These drugs target the voltage-operated Ca^2+^ channel (VOCC) and l-type Ca^2+^ channel (Cav1.1-1.4) in the smooth muscle by blocking Ca^2+^ influx and lowering cytoplasm Ca^2+^ concentration [10]. However, some problems result from these drugs such as low efficacy and many serious side effects like dysrhythmia, myocardial infarction and cancer [8,11,12], which led researchers to explore new leads from natural sources.

Pine needle was regarded as an anti-hypertension herb medicine in both ancient eastern and western countries. The medical effects of pine needle had been well documented in many studies and the pine related functional food, Pycnogenol^®^ has been developed to relief hypertension symptoms [13]. A study demonstrated that the ethyl acetate layer of *Pinus densiflora* and its self-fermented needle extract exhibited a vascular relaxation effect in isolated mouse aortic strip model [14]. Other experiments showed that the vascular relaxation effect of pine needle maybe mediated through various mechanisms, such as antioxidant scavenging effects or inhibition of catecholamine release [15,16].

The efficiency of current CCBs is still far from expectation and different hypertension patients usually respond differently depending on causes and progress, making it difficult to find a uniform treatment [17,18]. Despite the vascular relaxation effect of pine needle extract mentioned above, the detailed chemical compositions of pine needle and the vasodilator compounds remains unclear. In this study, we analyzed the chemical composition of the needle extract of 5LP by LC/MS/MS. The CCB activities in 5LP extract were evaluated via cytosolic Ca^2+^ fluorescent signals in A7r5 cell (a *Rattus norvegicus* aorta smooth muscle cell). Furthermore, the CCB candidates were characterized in the extracts and their ability to target calcium channels was also examined.

## 2. Results

### 2.1. Phenolic Composition in 5LP Needle Extract

**Figure 1:**
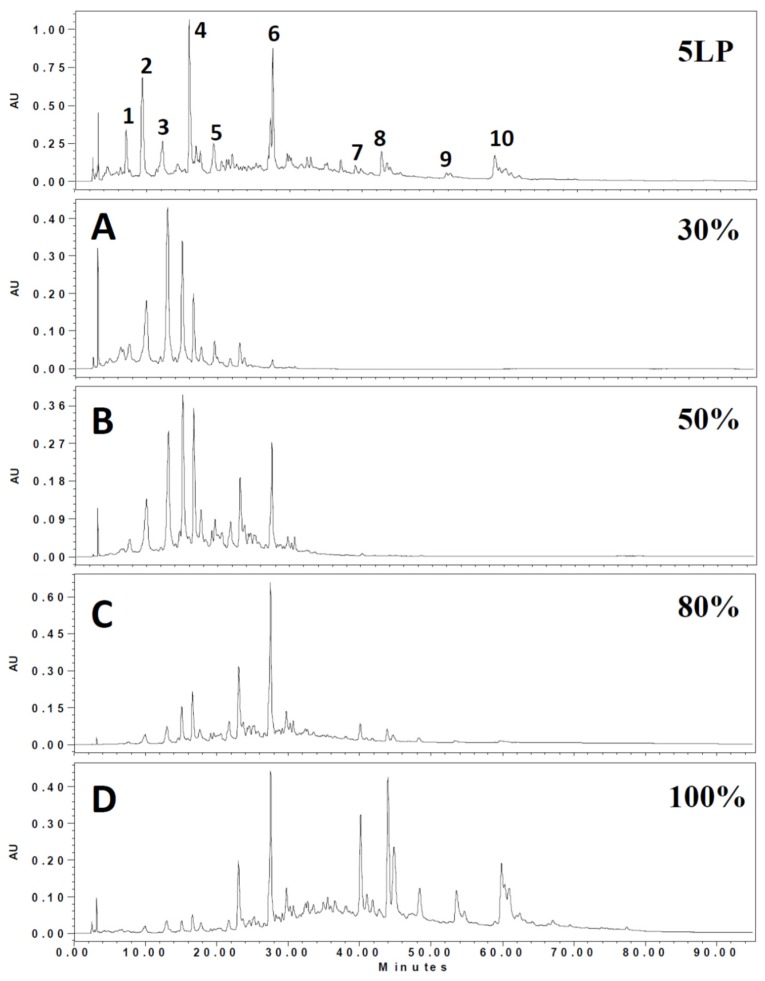
The ethyl acetate soluble components of 5LP needle were further fractionated by a Diaion HP-20 column with 30%, 50%, 80% and 100% methanol. Chemical constituents were analyzed and compared by HPLC (0–95 min).

To understand the phenolic composition of 5LP needles, LC/MS/MS were used to obtain phenolic profiles. Ten phenolics were tentatively identified and their retention times (RT), UV, molecular weights and fragmentation patterns are listed in Table 1 and Figure 1. Phenolic composition was mainly catechin (*m*/*z* 289), lignan glycoside (*m*/*z* 507) and flavonoid glycoside with a coumaroyl acylation functional group (*m*/*z* 593, 609, 563, 739).

### 2.2. Evaluation of the CCB Candidates from the 5LP Fractions

**Figure 2:**
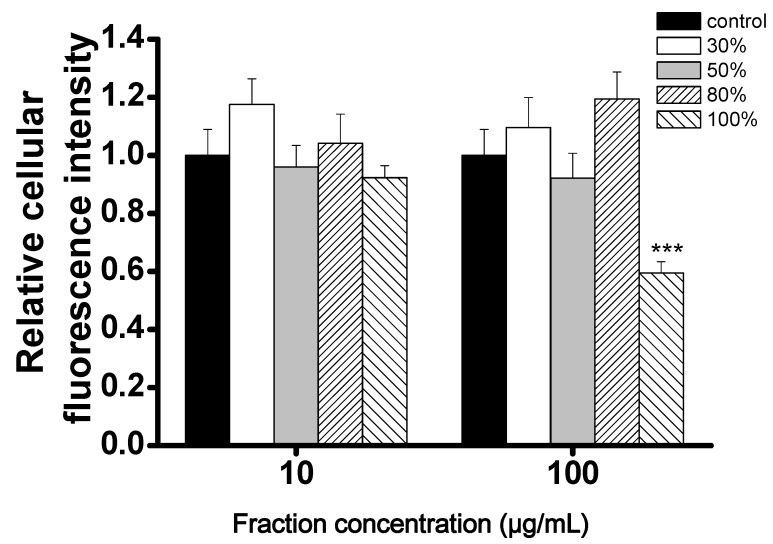
Reducing of intracellular Ca^2+^ levels in A7r5 cells treated with different 5LP Diaion HP-20 fractions. A7r5 cells were loaded with Fluo4-AM prior to incubation with various concentrations of Diaion HP-20 fractions (A:30%, B:50%, C:80%, D:100%). Intensity of fluorescence was collected at 0 and 30 min. All fractions were tested under two different concentrations, 10 and 100 μg/mL. Mean ± S.D. (indicated by vertical lines) was calculated from three different experiments. *** *p* <0.001 versus control values.

**Figure 3:**
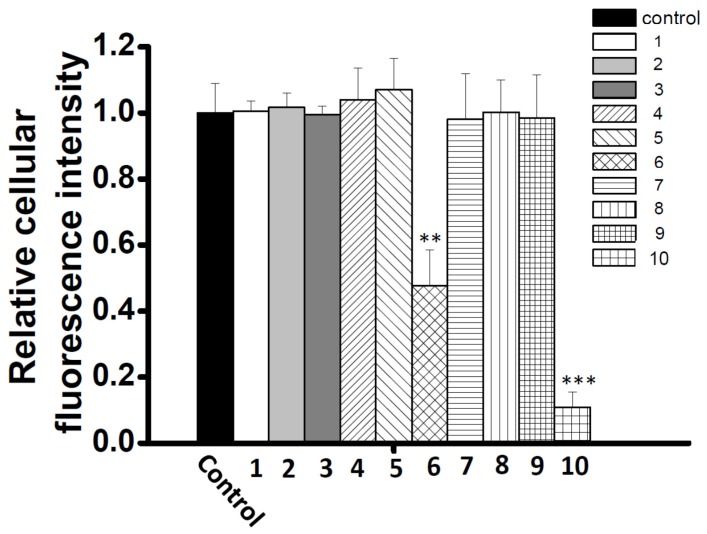
Reducing of intracellular Ca^2+^ levels of A7r5 cells treated with subfractions D1–D10 (0.3 mg/mL) fractions. A7r5 cells were loaded with Fluo4-AM prior to incubation with different fractions. Intensity of fluorescence was collected at 0 and 30 min. Mean ± S.D. (indicated by vertical lines) values were calculated from three different experiments. ** *p* <0.01 and *** *p* <0.001 versus control values.

**Figure 4:**
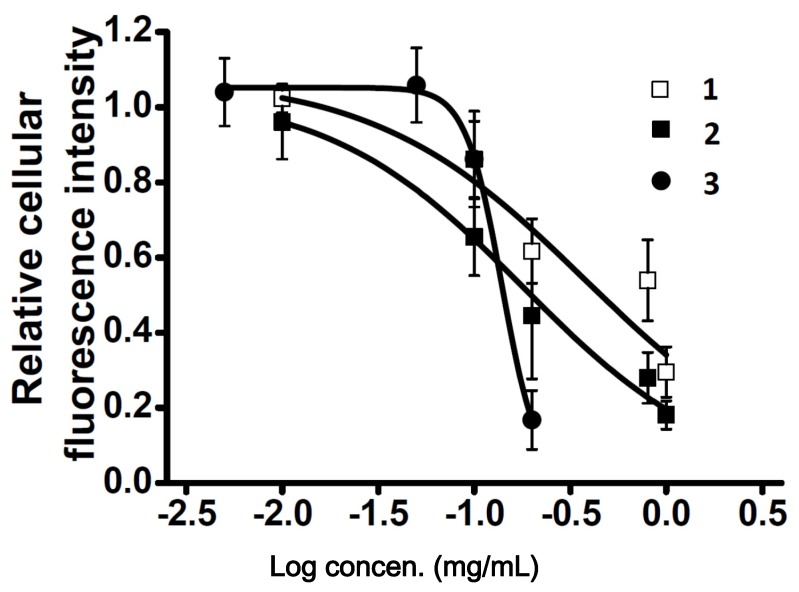
The inhibitory potency of compounds **1**–**3** on the relative cellular calcium level in A7r5 cells in normal HBSS solution. A7r5 cells were loaded with Fluo4-AM prior to incubation with various concentrations of compounds (compounds **1** and **2** at 1, 0.8, 0.2, 0.1 and 0.01 mg/mL; compound **3** at 0.2, 0.1, 0.05 and 0.005 mg/mL). Intensities of fluorescence were recorded at 0 and 30 min. The inhibitory potency was determined by calcium fluorescence intensity. Each curve represents the mean ± S.D. (indicated by vertical lines) and was calculated from three different experiments.

To explore new CCB candidates responsible for antihypertension treatment, cellular Ca^2+^ content was estimated by Ca^2+^ fluorescence signal intensity and Fluo4-AM in A7r5 cells. The ethyl acetate layer was passed through a Diaion HP-20 column to afford four fractions A–D (Figure 1), and then, these fractions were subjected to the Ca^2+^ fluorescence signal assay. Fraction D showed the most significant inhibition effect in cellular Ca^2+^ content (0.43 ± 0.13) compared to other fractions (Figure 2). Fraction D was further purified by HPLC to produce subfractions D1–D10 and these subfractions were assayed for bioactivity (Figure 3). Only subfractions D6 and D10 (at 0.3 mg/mL) displayed inhibition ratios of 0.48 ± 0.11 and 0.11 ± 0.05, and were further purified by TLC and HPLC. Finally, two lignans **1** and **2** were acquired after separation of subfraction D6 by TLC with dichloromethane:methanol (9:1). In addition, one acylated flavonoid glycoside **3** was isolated from subfraction D10 by HPLC. Quantitatively, the IC_50_ (50% inhibitory concentration) values of **1**–**3** were approximately 0.71, 0.36, and 0.20 mM, respectively. Inhibition activities were similar for these three purified compounds, however, the inhibition curve pattern of **3** is distinct from those of **1** and **2** (Figure 4). The structures of **1**–**3** are discussed below.

### 2.3. Characterization of the Vasodilation Candidates **1**–**3**

**Figure 5:**
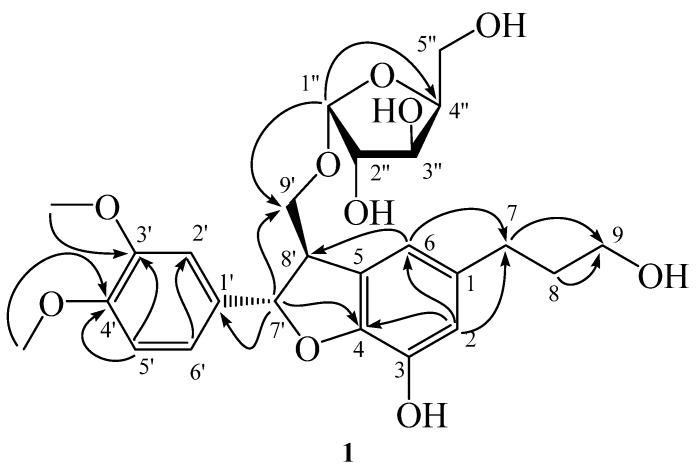
Structure and significant HMBC correlations of compound **1**.

Compound **1** was isolated as an optically active yellow powder with mp 163–165 °C. The high resolution electrospray ionization mass (HR-ESI-MS) analysis of **1** showed a pseudomolecular ion peak at *m*/*z* 515.1890 indicating the molecular formula as C_25_H_32_O_10_. The UV spectrum exhibited absorption maxima at 281, 236, and 219 nm, and IR absorption bands at 3446 and 1653 cm^−1^ were characteristic for the hydroxy and carbon–carbon double bond functionalities. In the ^1^H NMR spectrum of **1**, there were characteristics for four methylene signals at *δ* 1.79 (2H), 2.56 (2H), 3.56 (2H), 3.75 (1H) and 3.85 (1H), two methine protons at *δ* 3.56 and 5.53, two mutually meta-coupled aromatic protons at *δ* 6.56 and 6.58 and three aromatic protons of ABX coupling system at 6.91, 6.99 and 7.04, respectively. These signals suggested that **1** possessed a neolignan skeleton [25]. In addition, the ^1^H- and ^13^C-NMR spectra also exhibited typical signals at *δ*_H_ 3.64, 3.75, 3.86, 3.95, 4.02, 4.94, and *δ*_C_ 63.1, 78.8, 83.6, 85.8, 109.4, which were identified as an arabinose substituent [26,27]. The configuration of the sugar moiety was inferred from the ^1^H NMR coupling constants and the nuclear Overhauser effect spectroscopy (NOESY) spectrum. In the heteronuclear multiple bond correlation (HMBC) spectrum (Figure 5), the ^3^*J*-HMBC correlations from *δ* 4.94 (H-1″) to *δ* 85.8 (C-4″) and *δ* 65.2 (C-9′) revealed the presence of arabinofuranoside moiety and the connection of arabinofuranoside at C-9′, respectively. The other ^2^*J*- and ^3^*J*-correlation signals in the HMBC spectrum from H-8 to C-9 and C-1; from H-7 to C-9 and C-1; from H-9 to C-7 and C-8; from H-8′ and H-9′b to C-7′; from OCH_3_-3′ and -4′ to C-4′ and C-3′; from H-1′’ to C-3′’; from H-7′ to C-8′, C-9′, C-2′, C-6′, C-1′ and C-4; from H-2 to C-7, C-6 and C-3; from H-6 to C-7, C-8′, C-2 and C-4; from H-5′ to C-1′, C-4′ and C-3′; from H-6′ to C-7′, C-2′ and C-4′; from H-2′ to C-7′, C-6′ and C-4′, respectively, constructed the complete planar structure. The electronic circular dichroism (ECD) spectrum of **1** showed a negative Cotton effect at 223 nm (Δ*ε* − 0.59), which indicated the absolute configurations of **1** to be 7′*R* and 8′*S* [25]. Conclusively, the structure of **1** was characterized as (7′*R*,8′*S*)-7′-(3′,4′-dimethoxyphenyl)-3-hydroxy-8′-hydroxymethyl-7′,8′-dihydrobenzofuran-1-propanol 9′-*O*-α-l-arabinofuranoside, and hence, **1** is a new compound reported from natural sources for the first time and trivially named as pinumorrisonide A.

**Figure 6:**
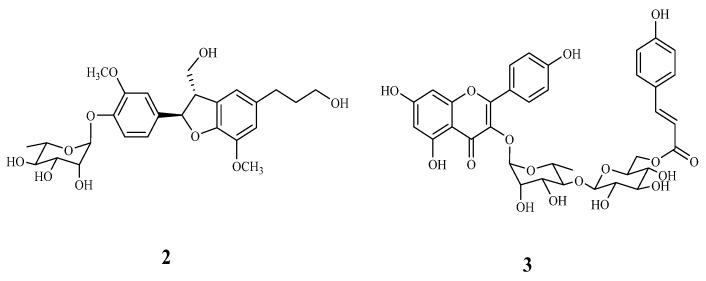
Structures of known compounds **2** and **3**.

Compound **2** was identified as icariside E4 by comparison of its physical and spectral data with those reported [25]. Comparatively, **2** showed a positive Cotton effect at 224 nm (Δ*ε* + 1.25), which was assigned the absolute configurations as 7′*S* and 8′*R* [25]. Compound **3** exhibited pseudomolecular signals with *m*/*z* 739 and fragment ions at *m*/*z* 593, 485, and 285 in its MS and MS/MS analytical spectra and tentatively identified as kaempferol coumaroyl-glucose-rhamnoside [24]. Its chemical structure was confirmed as kaempferol 3-*O*-α-(6‴-*p*-coumaroylglucosyl-β-1,4-rhamnoside) by comparison of its physical and spectral data with those reported [26]. The chemical structures of the known compounds **2** and **3** are shown in Figure 6.

### 2.4. Effects of Co-administration Vasodilation Candidates with Different Ca^2+^-elevation Agents

**Figure 7:**
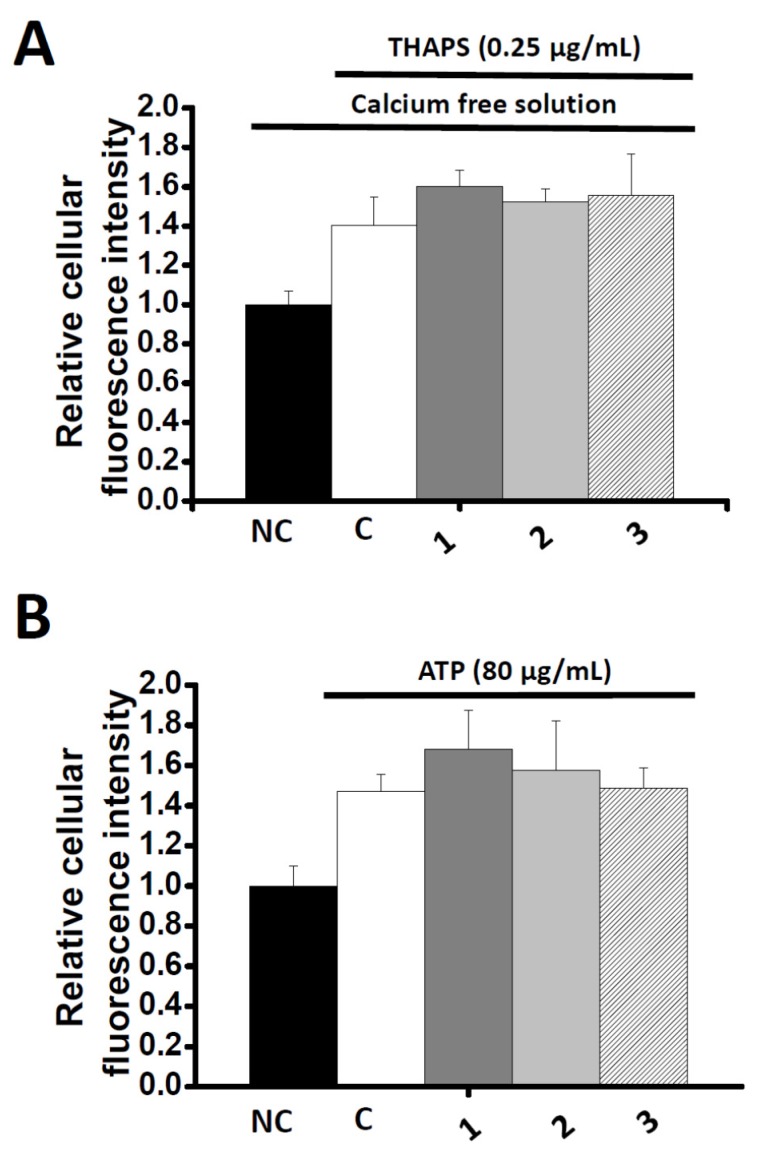
Effects of compounds **1**–**3** (200 μg/mL) on the calcium fluorescent intensity induced by (**A**) THAPS (0.25 μg/mL) with calcium free solution (10 mM EGTA) or (**B**) ATP (80 μg/mL) with normal HBSS solution. A7r5 cells were loaded with Fluo4-AM prior to incubation with different compounds. Intensity of fluorescence was collected at 0 and 30 min. Each bar represents the mean ± S.D. (indicated by vertical lines) and calculated from three different experiments.

**Figure 8:**
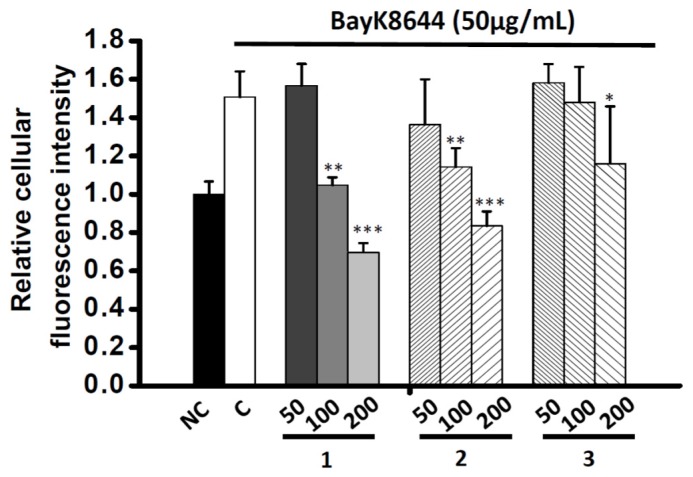
Effects of compounds **1**–**3** on the calcium fluorescent intensity induced by Bayk8644 (50 μg/mL). The compounds were tested under three different concentrations, 50, 100 and 200 μg/mL. A7r5 cells were loaded with Fluo4-AM prior to incubation with different compounds. Intensity of fluorescence was collected at 0 and 30 min. Each bar represents the mean ± SD (indicated by vertical lines) and calculated from three different experiments. * *p* <0.05, ** *p* <0.01 and *** *p* <0.001 versus control values.

In order to understand the mechanism of the Ca^2+^ inhibition activity of the vasodilation compounds, three Ca^2+^-elevation agents including thapsigargin (THAPS), adenosine triphosphate (ATP) and bayK8644 were co-administrated with compounds **1**–**3**. In the first set of experiments, THAPS induced Ca^2+^ storage release from the endoplasmic reticulum (ER). The depletion of Ca^2+^ pool evoked the cytosolic Ca^2+^ concentration even in the absence of extracellular Ca^2+^ (10 mM EGTA). THAPS (0.25 μg/mL) altered the fluorescence intensity ratio to 1.40 ± 0.14 compared to control. However, no obvious inhibition existed after co-incubating THAPS with 200 μg/mL of all compounds after 30 min (1.52 ± 0.07, 1.60 ± 0.08 and 1.55 ± 0.21, respectively) (Figure 7A). In another experiment using a receptor-operated Ca^2+^ channel (ROCC) agonist, ATP was administrated and fluorescence intensity significantly increased compared to control (1.47 ± 0.08). However, no obvious inhibition activity occurred (**1**, 1.67 ± 0.20; **2**, 1.57 ± 0.25; **3**, 1.49 ± 0.10, respectively) (Figure 7B). A voltage-operated Ca^2+^ channels agonist (VOCC), bayK8644, was serially diluted into 50, 100 and 200 μg/mL and co-incubated with all compounds. The Ca^2+^ fluorescence intensity was significantly reduced under 100 μg/mL of **1** and **2**, and 200 μg/mL of **3** (Figure 8).

## 3. Discussion

In this study, the phenolic composition of 5LP needle was analyzed by LC/MS/MS. The major phenolic components are catechin, lignan glycosides and flavonoid glycosides with acylation group. It has been reported that the major flavonoid aglycones in other *Pinus* species were kaempferol and quercetin. The loss of 162 and 146 mass units corresponded to the glucosyl and rhamnosyl residues, respectively. However, *m*/*z* 146 could be also designated to the coumaroyl moiety [27]. Thus, they were tentatively identified as mono or di-glycosides acylated with coumaric acid. These types of phenolic compounds have been proven to possess anti-aging effects in recent studies [28,29]. Francisco J. Luna-Vázquez et al. published a review article and listed more than two hundred different vasodilator compounds obtained from various plants. Phenolic compounds play a major role in all different categories and many of them had been studied under various examinations [30]. Researchers had also demonstrated that the French maritime pine (*Pinus pinaster*) extract, Pycnogenol^®^, augments vasodilatation effects in a two-week clinical trial [31]. The composition of Pycnogenol^®^ is mainly catechins, flavonoids and taxifolin [32,33,34,35]. Even though the health effect of pine extract has been well studied, there is little understanding about the detailed mechanism. Our experiment demonstrated that the ethanol extracts of 5LP needle have CCB effects and lowered cytosolic Ca^2+^ concentrations. Compounds **1**–**3** were further characterized and displayed significant cytoplasmic Ca^2+^ inhibition effects. Both of compounds **1** and **2** belong to dihydrobenzofuran lignan with arabinose and rhamnose moiety, respectively. Compound **2** had been discovered in *Juniperus communis*, *Tabebuia roseo-alba* and *Pinus densiflora* and exhibited anti-inflammatory, neuroprotective and antinociceptive activity [25,28,29,36]. Compound **1** was a new lignan glycoside reported from a natural source for the first time. It had a benzofuran skeleton similar to that of **2** with one arabinose moiety substituted at C-9′ position and one OCH_3_ group at C-4′ position. Compound **3** belongs to the acylated flavonoid glucoside and recently some acylated flavonoid have been reported, by our team, to have the ability to induce growth hormone release in an anterior pituitary cell model [37].

Cytoplasmic Ca^2+^ concentrations of smooth muscle cells are highly associated with vascular tone [8]. The reduction of Ca^2+^ concentration causes relaxation of contractile filaments followed by vasorelaxant action [38]. Two major types of receptors directly regulate the extracellular Ca^2+^ entrance, one is voltage operated Ca^2+^ channel (VOCC) and the other one is receptor-operated Ca^2+^ channel (ROCC) [8]. In normal HBSS condition, both of bayK8644 and ATP significantly induced the Ca^2+^ fluorescence signal after 30 min incubation. However, only bayk8644-induced fluorescence signals were inhibited by the three examined compounds. Despite all three phenolics having similar IC_50_ values, their inhibition curve patterns are quite different as compared two dihydrobenzofuran lignans (**1** and **2**) and acylated flavonoid glucoside (**3**). Moreover, the same phenomenon also appears in the inhibition assay with bayk8644, indicating that the drug binding sites on VOCC vary depending on their chemical structures. For the three prototypes of VOCC blockers, BTZ, DHP and PAA, the effect was mainly on the α1 subunit, at the third and fourth repeating domain between the fifth and sixth segments [39]. Some exceptions are on α2δ subunit which allosterically inhibit calcium flow [8]. Therefore, the complexity of the VOCC binding pocket alone with phenolic structures provides different choices for developing novel classes of Ca^2+^ inhibitor. In view of other reports, resveratrol increased cytoplasmic Ca^2+^ concentration through depletion of endoplasmic reticulum (ER) Ca^2+^ storage [38,40]. The effect of the three phenolic compounds on ER Ca^2+^ storage needs to be further investigated. In this regard, thapsigargin (THAPS), a sarco/endoplasmic reticulum Ca^2+^ ATPase (SERCA) blocker that increases cytoplasmic Ca^2+^ concentration was co-administrated with the three phenolic compounds under Ca^2+^-free external solution. Since there was no Ca^2+^ present in the extracellular areas, the refilling of Ca^2+^ only comes from the ER storage. Our experimental data showed that the THAPS-induced Ca^2+^ increase cannot be abolished by the three compounds. There is no other evidence that these compounds can pass through cell membrane and interact with the ER.

## 4. Materials and Methods

### 4.1. Chemicals and Materials

All chemicals were purchased from E. Merck Co. (Merck KGaA, Darmstadt, Germany) unless stated otherwise. HPLC grade acetonitrile was purchased from Fisher Scientific (Fair Lawn, NJ, USA). Acetic acid (99.7%) was obtained from J. T. Baker (Mallinckrodt Baker, Inc., Phillipsburg, NJ, USA). Dulbeco’s Modified Eagle Medium (DMEM), Hank’s Balanced Salt Solution (HBSS) and fetal bovine serum (FBS) were purchased from GIBCO (Grand Island, NY, USA). Fluo-4-AM was purchased from Invitrogen (Burlington, ON, Canada). Purified water was afforded by a Millipore clear water purification system (Direct-Q, Millipore, Billerica, MA, USA). The shoots of *P. morrisonicola* Hayata (5LP) were freshly obtained from the local pine tree farmer in Puli, Nantou, Taiwan and were authenticated by Dr. Nan-Hei Lin. The voucher specimens were deposited at the herbarium of Graduate Institute of Biotechnology, National Chung-Hsing University. The needles were detached from the shoots and washed with tap water, air dried in 40 °C oven for 2 days and stored at 4 °C before the extraction.

### 4.2. Preparation of Pine Needle Extract

Pine needles (1 kg) were ground into fine powder and extracted with 75% ethanol of 2 L at room temperature for three days. The extract was separated from the needles by filtration and partitioned with *n*-hexane and ethyl acetate successively. The ethyl acetate layer was subjected to LC/MS/MS analysis for phenolic constituents.

### 4.3. LC/MS/MS Analysis

The LC/MS/MS analysis was performed by Shimadzu LC-MS 8040 (Shimadzu Co., Kyoto, Japan) using a Shim-pack XR-ODS II column (2.0 × 100 mm, inner diameter 2.2 µm, Shimadzu Co., Kyoto, Japan). The column was maintained at room temperature, and 5 µL of sample was injected to the LC system at a flow rate of 0.2 mL/min. The mobile phase was composed of (A) acetonitrile and (B) 0.5% acetic acid in water. The gradient was as follows: 0–15 min, linear gradient from 5% to 15% A; 15–20 min, linear gradient from 15% to 20% A; 20–25 min, maintained at 20% A; 25–30 min, linear gradient from 20% to 25% A; 30–50 min, linear gradient from 25% to 30% A; 50–65 min, linear gradient from 30% to 40% A; 65–75 min, linear gradient from 40% to 60% A; 75–80 min, maintained at 60% A; 80–90 min, linear gradient from 5% A. For continuous sample analysis, the column was equilibrated with 6% B for 15 min before the next sample injection. The wavelength of UV absorbance was monitored at 280 nm.

The MS spectra (negative ion mode) were obtained on the Shimadzu LC-MS 8040 triple quadruple mass spectroscopy with an ESI interface. Ionization voltage was −4.5 kV, and source temperature was 250 °C. The flow rates of nebulizer and drying gas offered by nitrogen gas were 3 and 15 L/min, respectively. Argon gas was used as collision gas for tandem mass spectrometric experiment. All of MS spectra and data were collected and processed by the LabSolutions software (Version 5.60 SP2, Shimadzu).

### 4.4. Intracellular Ca^2+^ Imaging

A7r5 (a *Rattus norvegicus* aorta smooth muscle cell) were obtain from the American Type Culture Collection (ATCC, CRL-1444; Rockville, MD, USA). A7r5 cells were sub-cultured in DMEM containing 10% FBS. The cell culture condition was set at 37 °C with 5% CO_2_. For fluorescence imaging, cells were plated in 35 mm cell glass bottom culture dishes (World precision instruments Ltd., London, UK) and grown to 80% confluence (approximately 48 h).

The method for detecting the intracellular Ca^2+^ imaging was the same procedure as described previously [40,41]. Briefly, 80% confluence of the intracellular Ca^2+^ level of A7r5 cells was tracked and visualized by a preloaded fluorescent Ca^2+^-sensitive dye, Fluo4-AM. Cell-permeable Fluo4-AM was dissolved in DMSO to a concentration of 3 mM, and then further diluted to 3 μM in DMEM. The cell was washed three times with Hanks’ balanced salt solution (HBSS), and added with DMEM supplemented with 3 μM Fluo4-AM and 1% pluronic F127 (Sigma-Aldrich, St. Louis, MO, USA) for 30 min in a humidified 5% CO_2_ incubator at 37 °C. After washed three times with HBSS, cells were added with different reagents. The data was collected at two time points, 0 and 30 min. For experiment in extracellular Ca^2+^ free assay, 10 mM EGTA (Sigma-Aldrich, St. Louis, MO, USA) was added in the HBSS. Time-lapse images of live cells loaded with Fluo4-AM were collected by the IX71 inverted microscopy (Olympus, Tokyo, Japan). Fluctuation of fluorescence intensity of A7r5 cells treated with pine extracts were analyzed frame by frame with a time series analyzer [41,42]. This plug-in was used to analyze time-lapse image stacks. Cells were chosen as a region of interest (ROI) through mouse click and its fluorescence intensity of each time point was measured.

### 4.5. Isolation of Vasodilatation Candidates from Pine Needle Extract

The ethyl acetate layer of pine needle extract was chromatographed on a Diaion HP-20 column (GE Healthcare, Uppsala, Sweden) and eluted by water and step gradient with methanol (7:3, 5:5, 2:8, and 0:10) to afford fractions A–D, respectively. The isolated fractions A–D were subjected to the bioactivity examinations and only fraction D displayed the vasodilator effect, therefore it was further purified by HPLC. For HPLC purification, the sample was filtered through a 0.45 μm polyvinylidene difluoride (PVDF) membrane filter (PALL Corporation, Glen Cove, NY, USA) before passing through the liquid chromatography system coupled to a Model 600E photodiode array detector and Waters fraction collector III (Waters Corporation, Milford, MA, USA). Isolation was performed using a 250 mm × 4.6 mm i.e., 5 μm, Mightysil RP-18 GP column (Kanto Chemical Co., Tokyo, Japan). The mobile phase consisted of (A) acetonitrile and (B) water containing 0.5% acetic acid. The linear gradient was programmed as the follows: 0 min, 10% A; 5 min, 15% A; 15 min, 20% A; 25 min, 30% A; 55 min, 40% A; 70 min, 60% A; 85 min, 40% A; 90 min, 20% A; 95 min, 10% A. The column was maintained at room temperature and at a flow rate of 1 mL/min. The UV absorbance detection wavelength was set at 280 nm. The purified subfractions (D1–D10) were assayed for the bioactivity and the active fractions (D6 and D10) were further purified by the thin-layer chromatography (TLC) with dichloromethane and methanol (9:1) and HPLC with solvent mixture of A and B (40:60) to obtain highly purified compounds **1**–**3** for further experiments.

### 4.6. Spectroscopic and Spectrometric Studies of Purified Compounds

The melting point was measured using Yanaco MP-S3 micromelting point apparatus (Yanaco, Tokyo, Japan). Optical rotations were recorded on a Jasco P-2000 digital polarimeter (Jasco, Tokyo, Japan). The UV spectra were obtained by a Hitachi U-0080D diode array spectrophotometer (Hitachi, Tokyo, Japan). The IR spectra were examined with a PerkinElmer FT-IR Spectrum RX1 spectrophotometer (PerkinElmer, Waltham, MA, USA). NMR spectrum was acquired on a Bruker AMX500 (500 MHz) NMR (Bruker, Billerica, MA, USA) and a Jeol JNM-ECA600 (600 MHz) instrument (Jeol, Tokyo, Japan) at a constant temperature controlled and adjusted to room temperature, and chemical shifts are shown in *δ* values (ppm) with tetramethylsilane as an internal standard. ^1^H, ^13^C NMR and 2D spectra were recorded using methanol-*d*_4_ as the solvent. The ECD spectrums were analyzed on the Jasco J-815 spectropolarimeter (Jasco, Tokyo, Japan) with a 5 mm optical pathlength cell. The blank solvent spectra were measured with methanol. The scan ranged from 200–400 nm and was performed at room temperature. The data pitch is 1 nm with scan speed at 100 nm/min and accumulation of the data are the average from 5 scans. The ESI-MS and HR-ESI-MS were taken on a Bruker Solarix FT-MS spectrometer (Bruker, Billerica, MA, USA).

**Pinumorrisonide A (1)**: light yellow powder (CH_3_OH), m.p. 163–165 °C, [α]_D_^29^-12.1 (*c* 0.2, CH_3_OH); UV (CH_3_OH) λ_max_ (log ε): 281(3.37), 236(3.63), 219(3.62) nm; IR (KBr) ν_max_: 3446, 2950, 1653, 1559, 1022 cm^−1^; ECD (CH_3_OH, *c* = 6.30 × 10^−5^ M) 203 (Δ*ε* − 5.52), 211 (Δ*ε* + 2.36), 223 (Δ*ε* − 0.59), 240 (Δ*ε* +0.89), 295 (Δ*ε* +0.62); ^1^H-NMR (CD_3_OD, 500 MHz) δ 1.79 (2H, tt, *J* = 8.0, 6.5 Hz, H-8), 2.56 (2H, t, *J* = 7.5 Hz, H-7), 3.56 (1H, m, H-8′), 3.56 (2H, t, *J* = 6.5 Hz, H-9), 3.64 (1H, m, H-5″a), 3.75 (1H, m, H-5″b), 3.75 (1H, dd, *J* = 10.0, 6.0 Hz, H-9′b), 3.81 (3H, s, OCH_3_-3′), 3.81 (3H, s, OCH_3_-4′), 3.85 (1H, dd, *J* = 10.0, 4.0 Hz, H-9′a), 3.86 (1H, m, H-3″), 3.95 (1H, m, H-4″), 4.02 (1H, dd, *J* = 4.0, 2.0 Hz, H-2″), 4.94 (1H, d, *J* = 1.0 Hz, H-1″), 5.53 (1H, d, *J* = 6.0 Hz, H-7′), 6.56 (1H, br s, H-2), 6.58 (1H, br s, H-6), 6.91 (1H, d, *J* = 8.5 Hz, H-5′), 6.99 (1H, dd, *J* = 8.5, 2.0 Hz, H-6′), 7.04 (1H, d, *J* = 2.0 Hz, H-2′); ^13^C-NMR (CD_3_OD, 125 MHz) δ 30.8 (CH_2_, C-7), 35.8 (CH_2_, C-8), 53.3 (CH, C-8′), 56.6 (OCH_3_-3′), 56.6 (OCH_3_-4′), 62.3 (CH_2_, C-9), 63.1 (CH_2_, C-5″), 70.7 (CH_2_, C-9′), 78.8 (CH, C-3″), 83.6 (CH, C-2″), 85.8 (CH, C-4″), 88.7 (CH, C-7′), 109.4 (CH, C-1″), 110.8 (CH, C-2′), 113.0 (CH, C-5′), 116.2 (CH, C-6), 117.4 (CH, C-2), 119.4 (CH, C-6′), 129.1 (C-5), 136.6 (C-1′), 136.8 (C-1), 142.8 (C-3), 146.7 (C-4), 150.1 (C-4′), 150.6 (C-3′); ESI-MS (*rel. int.*): *m*/*z* 515 ([M + Na]^+^, 51), 381 (100), 353 (10); HR-ESI-MS *m*/*z* 515.1890 [M + Na]^+^ (Calcd. for C_25_H_32_O_10_Na, 515.1888).

## 5. Conclusions

In conclusion, we isolated three phenolic compounds from *P. morrisonicola* needle extract and identified as pinumorrisonide A (**1**), icariside E4 (**2**), and kaempferol 3-*O*-α-(6‴-*p*-coumaroylglucosyl-β-1,4-rhamnoside) (**3**) under the assistance of spectroscopic and spectrometric analyses. All of them showed inhibitory effects on the cytoplasmic Ca^2+^ concentration through VOCC in A7r5 cells. In addition, our results first indicated that *P. morrisonicola* could be a good candidate for further application as a new treatment for hypertension.

## Figures and Tables

**Table 1:** Phenolic compounds detected in the extract of five-leaf pine (5LP) needles ^a^.

Peak No.	RT (min)	λ_max_ (nm)	[M – H]^−^ (*m*/*z*)	MS^2^ (*m*/*z*)	Tentative Identification	Ref
1	21.4	294	651	489,325,163	Flavone-diglucoside	[19]
2	22.2	316	647	485,383,323	unknown	
3	24.5	280	289	245,205,203,137	Catechin	[20]
4	30.0	313	671	323,119	Dicaffeoyl-protocatechuic acid glucoside	[21]
5	34.1	281	509	491,361,313	Lignan glucoside	[22]
6	40.0	281	507	491,359,341,329	Icariside	[23]
7	67.5	267,314	593	447,285	Kaempferol coumaroyl-glucoside	[24]
8	67.8	267,314	609	433,300	Quercetin coumaroyl-rhamnose	[24]
9	75.2	268,316	563	417,284	Kaempferol coumaroyl-rhamnose	[24]
10	76.1	267,314	739	593,485,285	Kaempferol coumaroyl-glucose-rhamnoside	[24]

**^a^** The numbers of the compounds refer to the peak numbers in Figure 1.
